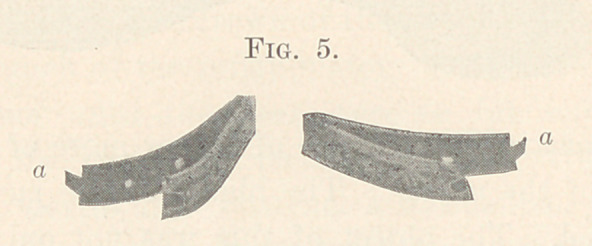# Ankylosis of the Jaws

**Published:** 1904-10

**Authors:** G. Lenox Curtis

**Affiliations:** New York


### ANKYLOSIS OF THE JAWS.[1]

BY G. LENOX CURTIS, M.D., NEW YORK.

My present purpose in speaking is to report some causes of the varieties in permanent ankylosis and to show plans of treatment that I have found very successful. I do this in the hope that it will be of service to others. Preparatory to the permanent cases, I will refer to cases of temporary ankylosis that I regard as unique and interesting.

Temporary ankylosis, so commonly found, can be so speedily treated successfully that little new remains to be told. Nevertheless these cases sometimes cause much trouble to both patient and practitioner, when they result in serious complications, which may occur if proper treatment is not given in the early stage. See Garretson, Marshall, and others for recognized methods.

The principal irritating causes of inflammation which lead to ankylosis of the jaws are exposed tooth-pulps, retarded, malposed, or impacted third molars, traumatism, cicatrix, tetanus, alveolar abscess, tonsillar, diphtheritic, and septic injections.

---

[1] Read at the annual session of the American Medical Association, Section on Stomatology, Atlantic City, June 7 to 10, 1904.

Permanent ankylosis is the result of osseous formations within the joint, causing partial or complete displacement or arrest of the synovial fluid, a condition, however, that may not occur for months or years of immobility. Fortunately this is rarely met with, except in cases of rheumatoid arthritis; because of the great activity of the lower jaw its joint is usually the last to become affected. Inflammatory conditions arising from any cause should be corrected as early as possible, in order to prevent cicatricial formations.

In one case of temporary ankylosis which had lasted for several days I found on examination that it seemed to be caused by an exposed pulp. This case was immediately relieved by extracting from the pulp a drop of blood and applying a dressing of campho-phenique.

The cicatricial variety follows suppurations and surgical operations through the face, such as are resorted to for the removal of tumors and necrosis of the jaws. When this condition is found in childhood and continues for a considerable length of time, it is generally followed by an arrest in the development of the face and jaws. In illustration of this are the photograph (Fig. 1) and casts

Fig. 1.

of the face and teeth of a boy, aged sixteen, who, when in his second year, fell from a window, fracturing his femur and also the inferior maxilla at the neck of the left condyloid process. The jaw fracture was not noticed until six months later, when the jaw was found to be ankylosed. The surgeon concluded that the trouble was due to muscular injury at the time of the fall. Thinking that in time the muscles would recover of themselves, he advised no treatment. Three years later another surgeon found the fractured jaw, but did not suggest any plan of relief. Later, indefinite different attempts were made to force the jaws apart, but were unsuccessful.

On examination, I found the ankylosis and the shortening of the jaws were due to the overlapping of the bones, which had become firmly fixed. The median line of the chin was considerably to the left. Several of the deciduous teeth which should have been cast off were present, and the mouth was in a generally disordered condition. I removed these teeth, reduced the inflammatory condition of the gums, and advised an operation for adjusting the ends of the fractured bones. I was told that several surgeons were consulted by the father, who was told that they would discourage surgical interference, consequently the boy was allowed to grow up in this unfortunate condition. My belief at that time was that the bones could be separated by means of a saw, or bur, and readjusted, and the ends of the fracture freshened and held in position until union of the bones was complete. He is now twenty-eight years of age.

Another case of ankylosis, the cause of which is of more than usual interest, is that of a young woman who for several years had been treated for repeated granular growths in the sockets of the lower left molars that had been extracted. On examination it was found that all of the jaw, including the ramus back of the first bicuspid, was necrosed. To my amazement I found the third molar was malposed and lying at the neck of the condyloid process directly below the condyle. The treatment consisted of opening the periosteum sufficiently to permit the removal of the tooth and the necrosed bone, and treating the wound until bone was reproduced. The periosteum was retained as an interosseous splint until sufficient new bone had formed to hold the jaw in position.

By this plan there was no shortening of the jaw and no deformity of the face. It is obvious that this operation was done within the mouth. I was unable to ascertain whether ankylosis on this side of the jaw was complete or was of the temporary variety. By the use of the screw-jacks a complete and permanent use of the jaw was re-established.

I saw in consultation another case of permanent ankylosis, resulting from a surgical operation made through the face for the removal of necrosed bone in the lower jaw, that resulted from an abscess on a molar. The cicatrix was several inches in length and about an inch in width. The patient told me he had been under treatment in a hospital for more than a year, much of which time his face was bandaged. I advised resecting of the scars, skin induction, and forcible separation of the jaw by means of screw-jacks.

I am now pleased to be able to bring before you here a patient who, at the age of fourteen, was brought to me in June, 1893. At the age of six years the patient had diphtheria, with extensive ulcers in the throat, the soreness from which continued for a considerable time after the disease had subsided. During this period pain was caused in opening the mouth, when the child was per-

Fig. 2.

mitted to take liquid food between the teeth. This method of taking nourishment became a habit. Four years later, owing to toothache, she was taken to a dentist, who, finding her jaws were ankylosed, referred her to a surgeon for treatment. Various methods, including the use of the "Grady screw," to pry and keep the jaws apart, were resorted to with slight results. Precaution was not taken, however, to protect the teeth from fracture, and some were broken and abscessed; gingivitis also resulted. Efforts to correct the ankylosed condition were finally abandoned, and the jaws closed and became rigid. At the time the effort was made to force the teeth apart the patient was encouraged to crowd solid food between the upper and lower teeth and crush it with the tongue against the roof of the mouth. This she was finally able to do with considerable success, but in doing this she had forced the lower teeth back and the upper teeth forward, causing some deformity. Fig. 2 shows a cast of the face. Examination showed the patient to be anæmic but otherwise in a fairly good state of health.

By forcing a wedge between the teeth I was able to secure one-eighth of an inch space on the left side. The reason for this was that on the left side there was only cicatricial ankylosis, while on the right side it was osseous. The condition of the mouth was deplorable. General gingivitis prevailed, and several of the permanent teeth were loose, while others were abscessed, and many of the deciduous teeth were present, thus retarding the full eruption of the permanent teeth. The crown of the upper right central was lost. By treatment much of the inflammatory condition of the gums was reduced, but not until the jaws were so far separated that the abscessed and the deciduous teeth could be extracted. My first thought was to devise and construct a mechanism that could force the jaws apart by causing an even pressure on the teeth. Fig. 3

Fig. 3.

shows the depressor made of steel spring, which I was able to crowd between the jaws while the wedges were in position and while the patient was under profound anæsthesia. While the head was firmly held by an assistant I was able to put sufficient force to the depressor to gain one-eighth of an inch space. With this space I was able to insert a flexible double screw-jack, represented by Fig. 4, that I

also devised for this purpose. The surface of the hand-depressor and the blades of the screw-jack were serrated, the object of which was to lessen the danger of slipping. If it is necessary to further reduce this danger, soft vulcanite rubber or gutta-percha may be

Fig. 4.

placed on the masticating surface of one or more of the molar teeth on either side of the mouth. The blades of the jack were made of thin spring steel. The object of this was not only to cause even bearing on the teeth, but to prevent undue pressure on the teeth and luxation of the jaws. The screws were purposely made long, so that the patient might tighten or loosen the jack at will. By this simple mechanism the patient was enabled to adjust it and make as slight or as much pressure as could be easily tolerated. This patient was able to wear this jack much of the time both day and night. When three-eighths of an inch space had been secured soft wax was flowed over the blades of the screw-jack and the jack was again put in position. By this means I was able to secure an impression of the antagonizing ends of the teeth, by which casts were made and splints of vulcanite were constructed, the approximating surfaces of which were made flat, so that when in place there was an equal bearing at all points. These splints enabled me to put greater pressure on the screw-jacks as well as eliminating all danger of fracturing the teeth. As the jaws opened, better fitting splints were applied. Chloroform was administered every few weeks, and all possible pressure was made to force the jaws apart. Almost from the beginning of the treatment there was an inflammation established in the right joint. While at times this operation was attended by considerable discomfort to the patient, which prolonged the work, it had much to do with the final success, because absorption of the osseous deposit within the joint was established, and by this constant agitation it continued until a fair action in the joint was established.

One of the things which retarded our efforts was the degenerated temporal and masseter muscles because of years of disuse. These muscles required redevelopment from that condition found in a child of six years to that of a young girl of sixteen. Until this was accomplished there was but moderate benefit derived from opening and closing the mouth. In order to develop the strength of the muscles of the face, as well as to elongate them, I devised a set of springs which were securely fastened in grooves cut in the approximating surfaces of the splints on either side of the mouth (Fig. 5). At the forward end of the grooves there was an opening

Fig. 5.

made through the splint of sufficient size to accommodate the studs *a, a,* which were one-sixteenth of an inch in thickness and one-eighth of an inch in length. The principal object of these studs was to prevent the springs from slipping out of place, and to doubly secure them they were also wired to the lower splint. These springs were very stiff, and only with great effort by the patient could be compressed. In order to get the splints into the mouth with the springs in position they were applied while bound tightly together, and when in position these ligatures were cut. At the end of one year's treatment the patient had about one-half the normal opening of the jaw, and for the next year the work of continuing the treatment was intrusted to her, because by personal illness I was absent from practice. On my return I was pleased to find that substantial progress had been made, the space gained was maintained, and that the muscles had materially improved. I took up the work again along the same lines and continued until almost the normal opening of the jaws had been secured, with, however, but little lateral motion, the adhesions which held the left side of the jaw readily giving way to the continued pressure of the jack. In the course of a year the patient's health demanded exclusive attention, and because of tuberculosis further maxillary irritation was at this time discontinued. Within the past six years, however, the patient has seldom found it necessary to make use of the springs; her health also has gradually improved. As you can see, the patient, though not robust, is in fairly good condition.

DISCUSSION.

*Dr. G. V. I. Brown,* Milwaukee.—I think Dr. Curtis is in danger of being misunderstood, since he evidently describes conditions of true and false ankylosis. He speaks of permanent and temporary ankylosis, and gives as etiologic factors malimposed third molars, pulpitis, and conditions of that character. What Dr. Curtis really means is not ankylosis, but trismus. I think we ought to draw a very distinctive line between a muscular contraction of a temporary nature, as described, due to more or less direct irritation of the nerve-trunks, and a condition caused by inflammatory processes or degenerative conditions of the temporo-maxillary articulation. So far as operative measures are concerned nothing can be said but the highest praise. These cases are extremely troublesome, and Dr. Curtis's results are a warrant that the proper methods were employed.

*Dr. Charles F. Allan,* Newburgh, N. Y.—I have never had a case of osseous formation in the jaw, and I think they are very, very rare. The coagulation of the secretions as a result of traumatism, which causes the ankylosis, is sufficient to make a strong bar to the jaws closing as they should. I have never found that any application made to cause absorption was in any way effective. Pressure under chloroform and daily use of the screw opener by the patient would be the only means to cause return to normal conditions.

## Figures and Tables

**Fig. 1. f1:**
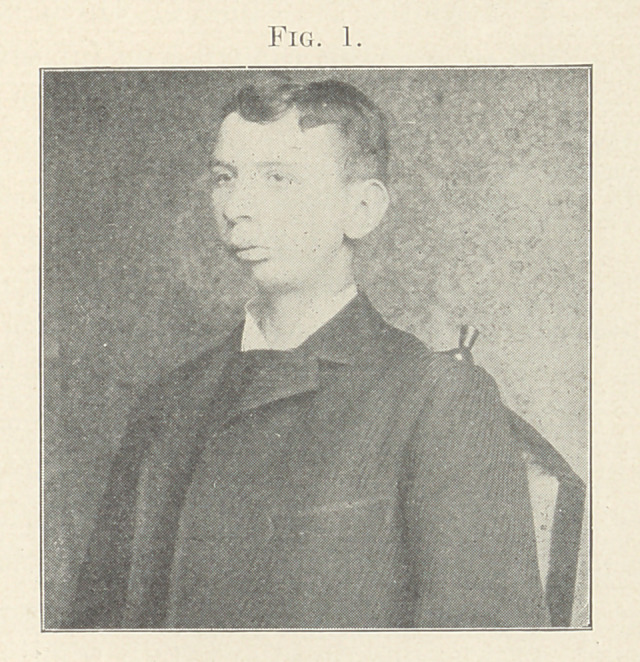


**Fig. 2. f2:**
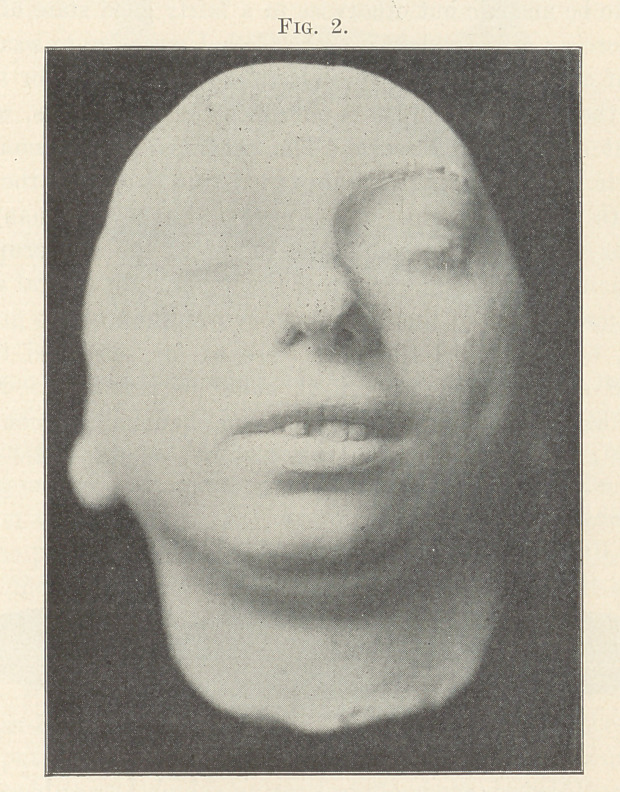


**Fig. 3. f3:**
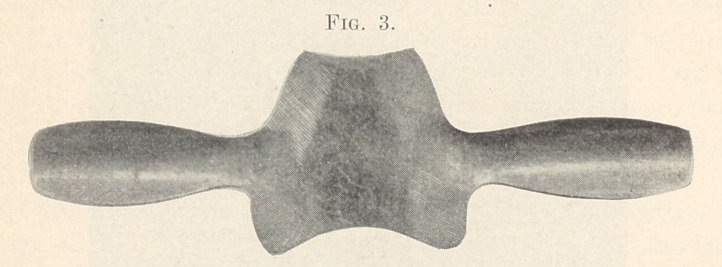


**Fig. 4. f4:**
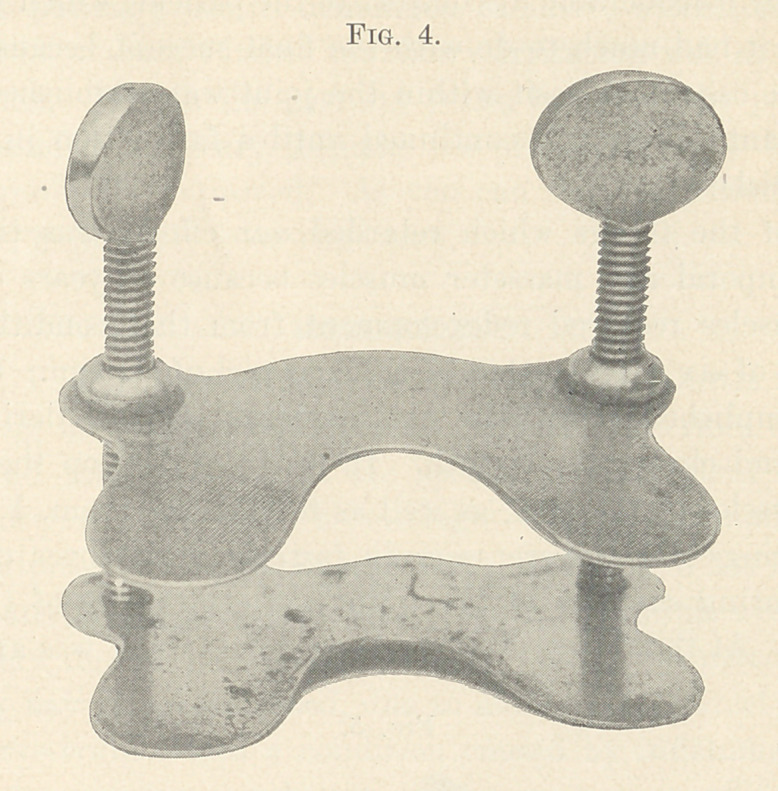


**Fig. 5. f5:**